# Na^+^/H^+^ exchanger NHE1 regulation modulates metastatic potential and epithelial-mesenchymal transition of triple-negative breast cancer cells

**DOI:** 10.18632/oncotarget.8520

**Published:** 2016-03-31

**Authors:** Schammim Ray Amith, Jodi Marie Wilkinson, Larry Fliegel

**Affiliations:** ^1^ Department of Biochemistry, University of Alberta, Edmonton, Alberta, Canada

**Keywords:** NHE1, metastasis, vimentin, epithelial-mesenchymal transition, triple-negative breast cancer

## Abstract

In triple-negative breast cancer (TNBC), the high recurrence rate, increased invasion and aggressive metastatic formation dictate patient survival. We previously demonstrated a critical role for the Na^+^/H^+^ exchanger isoform 1 (NHE1) in controlling metastasis of triple-negative cells. Here, we investigated the effect of changes to three regulatory loci of NHE1. Two via the Ras/Raf/ERK/p90^RSK^ pathway: p90^RSK^/14-3-3 (S703A) and ERK1/2 (S766,770,771A, SSSA) and a third via a calmodulin-binding domain (K641,R643,645,647E, 1K3R4E). MDA-MB-231 cells with a mutation at the p90^RSK^ site (S703A-NHE1) changed from a wild-type mesenchymal morphology to a smaller epithelial-like phenotype with a loss of expression of mesenchymal marker vimentin. S703A cells also had reduced metastatic potential and markedly decreased rates of migration, invasion, spheroid growth, anchorage-dependent and soft agar colony formation. Similarly, BI-D1870, a specific inhibitor of p90^RSK^, significantly inhibited the metastatic potential of highly invasive MDA-MB-231 and moderately invasive MDA-MB-468 TNBC cells, but was minimally effective in non-invasive Hs578T TNBC cells. In contrast, invasion and spheroid growth were unaffected in cells containing NHE1 with mutations interfering with its activation by ERK1/2 (SSSA), though rates of migration and colony formation were reduced. Cells with a constitutive activation of NHE1 via the 1K3R4E mutation exhibited higher rates of migration, invasion, and spheroid growth. Taken together, our data demonstrate the critical role of NHE1 in metastasis, and suggest a novel link between NHE1 and the expression and cytosolic organization of vimentin, a key factor in epithelial-mesenchymal transition, that is dependent on p90^RSK^/14-3-3-mediated activation of the exchanger.

## INTRODUCTION

Metastasis is the leading cause of mortality in patients with breast cancer, but prognoses are often poorest in those diagnosed with the triple-negative breast cancer (TNBC) subtype. TNBC is aggressively tumorigenic, with high recurrence rates that show limited response to chemotherapy [1, 2]. Since TNBC cells lack the expression of estrogen, progesterone, and human epidermal growth factor-2 (HER2) receptors, targeted therapies dependent on these receptors are ineffective. A complicating factor in cancer cells is that acid-base homeostasis becomes imbalanced at the inception of oncogenic transformation with an increasing intracellular pH (pH_i_) [3, 4]. This intracellular alkalinization of tumor cells and the resultant extracellular acidification of the tumor microenvironment drive malignancy [5, 6]. Malignant cancer cells lose the polarity and cell-cell contacts typical of differentiated epithelial cells, instead acquiring stem cell characteristics, and the motile, invasive mesenchymal phenotype that enables dissemination out of the primary tumor and into the bloodstream. This complex developmental process is called epithelial-mesenchymal transition (EMT). Cancer-associated EMT is reversible. This allows for the re-differentiation of invasive cells to an epithelial phenotype *via* mesenchymal-epithelial transition (MET), a process that promotes cell colonization and the formation of new metastases at secondary sites in the body distant from the primary tumor [7].

The identification of promising new targets is critical in the search for more efficacious and potent treatment regimes against TNBC. One of these targets is the Na+/H+ exchanger isoform 1 (NHE1). NHE1 is a ubiquitously expressed ion transporter found in all mammalian cells. It regulates pH homeostasis *via* the electroneutral exchange of one intracellular H^+^ for one extracellular Na^+^ ion [8] and is responsible for the elevation of pHi in TNBC cells and for extracellular acidification of the tumor microenvironment [5, 6]. We recently demonstrated that NHE1 inhibition increases the *in vitro* efficacy of paclitaxel chemotherapy in TNBC cells and decreases their viability, motility, and invasiveness. Also, deletion of NHE1 dramatically reduced xenograft tumor growth of TNBC cells *in vivo* in athymic nude mice [9]. The activation of NHE1 is regulated [10] and we therefore sought to elucidate the underlying regulatory mechanisms in TNBC cells that may influence metastatic behavior.

NHE1 has a transmembrane domain spanning amino acids 1-500. This region mediates ion flux, while the cytosolic C-terminal domain (aa 501-815) is essential for regulation of exchanger activity [11]. Regulation of NHE1 occurs through both protein binding and phosphorylation by various protein kinases (reviewed in [12]). Amino acids 636-659 span the region involved in the auto-inhibition of NHE1. Mutations to this region can prevent auto-inhibition of NHE1 and thus constitutively activate the protein [13]. Calmodulin, in complex with calcium, which binds to this portion of the C-terminal tail, also prevents NHE1 auto-inhibition [14]. The more distal region of the NHE1 C-terminal (aa 660-815) is key in its regulation *via* phosphorylation by various protein kinases [15, 16]. The activation of NHE1 by the Ras/Raf/ERK/p90^RSK^ pathway in particular is correlated with breast cancer progression and metastatic behaviour [17, 18]. In this pathway, one amino acid of interest on NHE1 is Ser^703^. Ser^703^ is phosphorylated by p90^RSK^, which stimulates activity in response to serum and, when phosphorylated, becomes a binding site for 14-3-3 regulatory proteins [18-20]. A second region of interest related to this pathway is the region around the group of amino acids Ser^766^, Ser^770^ and Ser^771^. These have been identified as ERK1/2 phosphorylation sites [21-23] and are also involved in activation of NHE1.

In the present study, we investigated the effect of regulatory modifications to NHE1 to determine their involvement in the migratory, invasive, and colony-forming capacity of TNBC cells. We examined three different regulatory mutations: two were on the phosphorylation sites Ser^703^ and the group of Ser^766^, Ser^770^ and Ser^771^; and the third was the high-affinity calmodulin-binding regulatory site of NHE1. We utilized MDA-MB-231 cells, representative of the metastatic triple-negative clinical subtype of breast cancer. These cells have a mesenchymal, invasive phenotype [24]. We replaced the endogenous NHE1 protein [9] with mutant NHE1 proteins: S703A, where serine 703 was changed to a non-phosphorylatable alanine; SSSA, where serine residues 766, 770 and 771 were altered to non-phosphorylatable alanine residues; and 1K3R4E, where positively-charged lysine 641 (1K) and arginine 643, 645 and 647 (3R) residues were replaced by negatively-charged glutamic acids (4E). This latter mutation interferes with auto-inhibition of the membrane domain and results in the constitutive activation of NHE1 [14, 25]. We found that S703A cells changed to a more epithelial-like phenotype, losing expression of the intermediate filament protein vimentin and migratory and invasive ability. The specific p90^RSK^ inhibitor of BI-D1870A mimicked effects on migration, invasion, and colony growth in other triple-negative breast cancer cells. Another NHE1 hyperactive mutation also made MDA-MB-231 cells more metastatic. Our data strongly suggest that Ser^703^ may be a critical phosphorylation switch that regulates the morphology and EMT/MET of the highly invasive triple-negative MDA-MB-231 breast cancer cells. Overall, this study presents the first evidence of a novel link between NHE1, its activity, and the regulation of EMT that is associated with the onset of breast cancer metastasis in TNBC cells.

## RESULTS

### Characterization of NHE1 expression and Na^+^/H^+^ exchange activity in wtNHE1 and mutant NHE1-expressing MDA-MB-231 breast cancer cells

**Figure 1:**
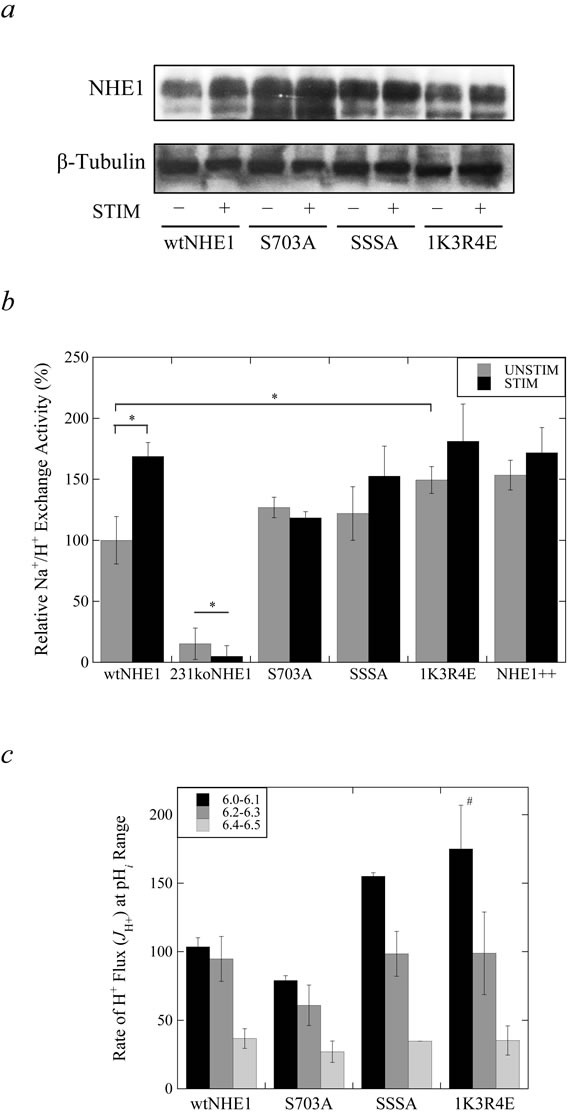
Characterization of wild-type and mutant NHE1 expression and Na^+^/H^+^ exchange activity in MDA-MB-231 cells **a.** Western blot analysis of cell lysates of MDA-MB-231 cells expressing wild-type and mutant NHE1 proteins. NHE1 protein expression was examined in wtNHE1, S703A, SSSA and 1K3R4E cells probed with anti-NHE1 antibody. Cells were grown in either serum supplemented (−, 10% serum, UNSTIM) or serum depleted (+, 0.2% serum, STIM) conditions for 24 hr. prior to whole cell lysis. One representative western blot with anti-NHE1 antibody and anti-tubulin antibody is shown. NHE1 expression from four independent experiments was quantified by densitometry and determined to be unchanged between wild-type and mutant NHE1 cells (Supplementary Figure 1a). **b.** and **c.** Determination of NHE1 activity in mutant and wild-type cells. An acute acid load was induced by treatment with ammonium chloride. The rate of Na^+^/H^+^ exchange was calculated from the slope of the first 20 sec. of recovery post-acid load and expressed as ΔpH*_i_*/sec. ΔpH*_i_*/sec values measured for each cell type were normalized to that of the unstimulated wtNHE1 control values. b, Relative Na^+^/H^+^ exchange rate of mutant NHE1 cells compared to wtNHE1, 231koNHE1, and NHE1++ cells where NHE1 is over-expressed, as described in the Materials and Methods. c, H^+^ flux of wild-type and mutant NHE1 cells. Cells were acidified to varying pH*_i_* and allowed to recover in NaCl-containing media. The rate of H^+^ flux (*J*_H_^+^) was calculated as a product of the buffering capacities of cells (*B* mmol • 1^−1^ • pH unit^−1^; data not shown) and the rate of change of pH*_i_* over time (ΔpH*_i_*/sec) [^#^*P* < 0.05, *N* = 6].

To investigate the effect of alterations to NHE1 regulation in triple-negative breast cancer cells, we replaced endogenous NHE1 with NHE1 protein containing mutations to various regulatory regions. MDA-MB-231 cells deficient in NHE1 (231koNHE1) were described earlier [9]. We stably expressed wild-type NHE1 (wtNHE1) and mutant NHE1 protein into these cells with the following mutations to the cytosolic C-terminal domain: Ser^703^Ala (S703A), Ser^766,770,771^Ala (SSSA) and Lys^641^Arg^643,645,647^Glu (1K3R4E). We used western blot analysis of whole cell lysates of mutant NHE1 cell lines to verify expression in comparison to wtNHE1 cells. We examined NHE1 protein expression levels in cells grown in either serum-supplemented (10% serum) or serum-depleted (0.2% serum) culture media (Figure 1a). Densitometric quantification was relative to that of the loading control (β-tubulin). The results confirmed that NHE1 was stably expressed in the MDA-MB-231 cells and, additionally, no differences in protein expression were observed amongst cell types with varying serum concentrations (*P* > 0.05, *N* = 4, Supplementary Figure 1a).

We next examined the activity of the wild-type and mutant NHE1 proteins in MDA-MB-231 cells. Cells were subjected to 50 mM ammonium chloride-induced acidification for 3 min. and allowed to recover in the presence of NaCl. Figure 1b shows NHE1 activity relative to the wild-type in normal (UNSTIM) or reduced serum (STIM). Wild-type NHE1 protein was significantly (*P > 0.001, *N* = 6) activated by reduction in serum as reported earlier [9, 26]; loss of Na^+^/H^+^ activity in 231koNHE1 cells is shown for comparison. Neither the S703A nor the SSSA mutant proteins were significantly activated by reduction in serum, though there was a tendency towards an increase in the SSSA-expressing cells. The 1K3R4E mutant protein was significantly (*P > 0.001, *N* = 6) more active than the wild-type protein, comparable to Na^+^/H^+^ exchange rates observed in MDA-MB-231 cells over-expressing NHE1 (NHE1++), though this activity was not further increased by reduction of the serum concentration.

A more detailed analysis of NHE1 activity involved acidification of the various cell types to different pH*_i_* followed by a calculation of the rate of proton flux at varying ranges of pH*_i_* (Figure 1c). This analysis of NHE1 activity showed that increased Na^+^/H^+^ exchange activity of 1K3R4E cells corresponded to a higher affinity for H^+^ at an acidic pH*_i_* of 6.0-6.1 (^#^*P* > 0.05, *N* = 6, Figure 1c). We also used optical emission spectroscopy to compare ionic concentrations of Na^+^ and K^+^ to determine whether mutations to NHE1 regulation works by modulating intracellular and extracellular concentrations of these monovalent cations. In growth conditions where NHE1 is either stimulated by serum starvation (0.2% serum) or unstimulated (10% serum), a significant increase in intracellular K^+^ was observed in S703A cells compared to wtNHE1 cells regardless of stimulation (^#^*P* < 0.05, ^+^*P* < 0.01, *N* = 6, Supplementary Figure 1b). No other differences in intracellular or extracellular (in culture media) cation concentrations were observed between the wtNHE1 and mutant NHE1-expressing cells.

### Effect of mutations to NHE1 on MDA-MB-231 breast cancer cell morphology

**Figure 2:**
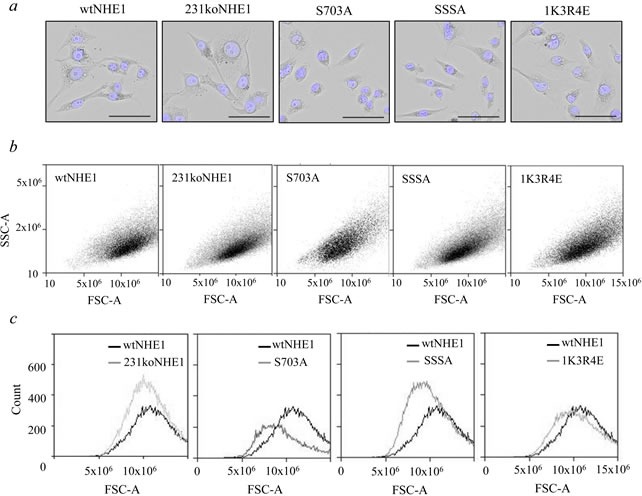
Comparison of morphology of mutant and wild-type NHE1-expressing MDA-MB-231 cells The morphology of mutant NHE1 cells was compared to wtNHE1 and 231koNHE1 cells by microscopy **a.** and flow cytometry (*b, c*). **a.** Bright-field images showing fixed cells with Hoechst nuclear staining (blue). Scale bars represent 50 μm at 20X magnification. **b.** and **c.** Quantification of differences in cell size and granularity. **b.** Dot plots showing cell size (FSC-A) *versus* granularity (SSC-A), and **c.,** histogram overlays of size *vs*. number of cells.

Replacement of the wild-type NHE1 with S703A-NHE1 protein caused noticeable changes in cell morphology that were examined in further detail. When adherent, fixed cells were visualized under bright-field microscopy, wtNHE1, 231koNHE1, SSSA, and 1K3R4E cells exhibited the stellate cell morphology typical of the mesenchymal parental MDA-MB-231 breast cancer cells [27]. In contrast, S703A cells exhibited a more rounded or cuboidal epithelial-like phenotype and the majority of cells appeared smaller than cells expressing wtNHE1 (Figure 2a). When cell size (Forward Scatter-Area, FSC-A) *versus* granularity (Side Scatter-Area, SSC-A) was analyzed by flow cytometry, S703A cells were significantly smaller than wtNHE1 cells (Figure 2b, 2c). SSSA cells were also slightly smaller than the wtNHE1, but the difference was not considered significant. There was no difference in size between wtNHE1, 231koNHE1, and 1K3R4E cells. The degree of granularity (a measure of cytosolic complexity) did not vary between the cell types.

### Effect of NHE1 mutations on the proliferation of MDA-MB-231 cells

**Figure 3:**
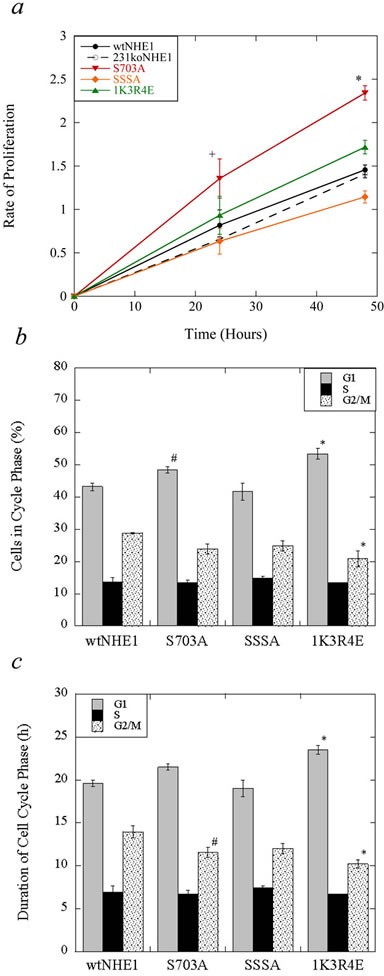
Cell proliferation and cell cycle analysis of mutant and wild-type NHE1-expressing MDA-MB-231 cells **a.** Measurement of cell proliferation rates over time. The rate of proliferation of wtNHE1, 231koNHE1, S703A, SSSA and 1K3R4E cells was assessed at 0, 24, and 48 hr. by spectrophotometric analysis of the enzymatic reduction of yellow tetrazolium MTT. Data shown are representative of mean OD values for each cell type at indicated time points compared to initial OD values at 0 hr. [+*P* < 0.01; *P < 0.001; *N* = 3]. **b.** and **c.** Cell cycle analysis of mutant NHE1 cells relative to wtNHE1 cells. Laser-scanning cytometry was used to quantify changes in cell cycle progression over 24 hr. as described in the Materials and Methods. **b.** Analysis of cell numbers in each phase of the cell cycle in wtNHE1, S703A, SSSA and 1K3R4E cells. The percentage of cells in each phase at a single time point (univariate analysis) of the cell cycle is calculated from mean integral fluorescence (which is proportional to DAPI-DNA binding) and mean maximum pixel values (which indicates chromatin concentration). [^#^*P* < 0.05, *P < 0.001 *vs*. control; *N* = 3]. **c.** Duration of cell cycle phases in wtNHE1, S703A, SSSA and 1K3R4E cells. The length of time spent in each phase of the cell cycle was estimated using the following equation: T_*Phase*_ = [T_C_ × ln(f_*Phase*_+1)]/ln2, where T_C_ is the duration of the cell cycle or population doubling time, and f_*Phase*_ is the fraction of cells in a particular phase of the cell cycle [59].

To investigate the effect of negating NHE1 activation mediated by p90^RSK^/14-3-3 (S703A) and ERK1/2 (SSSA), *versus* constitutively activating NHE1 by blocking exchanger auto-inhibition (1K3R4E), we compared the rates of proliferation between mutant NHE1 cells, cells expressing wild-type NHE1, and 231koNHE1 cells. As seen in Figure 3a, S703A cells had a significantly higher rate of proliferation compared to wtNHE1, 231koNHE1, and other mutant-containing cells at 24 and 48 hr. (^+^*P* < 0.01, *P < 0.001, *N* = 3). We then used laser-scanning cytometry to analyze potential differences in the progression of mutant NHE1 and wtNHE1 cells through the cell cycle. We quantified the percentage of cells in each phase of the cell cycle at a single time point 24 hr. after an equal number of cells were plated in serum-supplemented media. Here, mean integral fluorescence is proportional to DAPI-DNA binding and maximum pixel values indicate chromatin concentration [28]. We found that there were significantly more S703A cells in the first growth phase G1 compared to wtNHE1 cells (^#^*P* < 0.05, Figure 3b), while the estimated duration of the second growth and mitotic phases (G2/M) for S703A cells was much shorter than in wtNHE1 cells (^#^*P* < 0.05, Figure 3c). We also found that there were more 1K3R4E cells in G1, but fewer cells in G2/M. Similarly, the duration of G1 was longer and G2/M was shorter, respectively, in 1K3R4E cells relative to wtNHE1 cells (*P < 0.001, Figure 3b, 3c). Progression of SSSA cells through the cell cycle was comparable to wtNHE1 cells.

### Effect of mutations to NHE1 regulatory regions on migratory and invasive capacity of MDA-MB-231 cells

**Figure 4:**
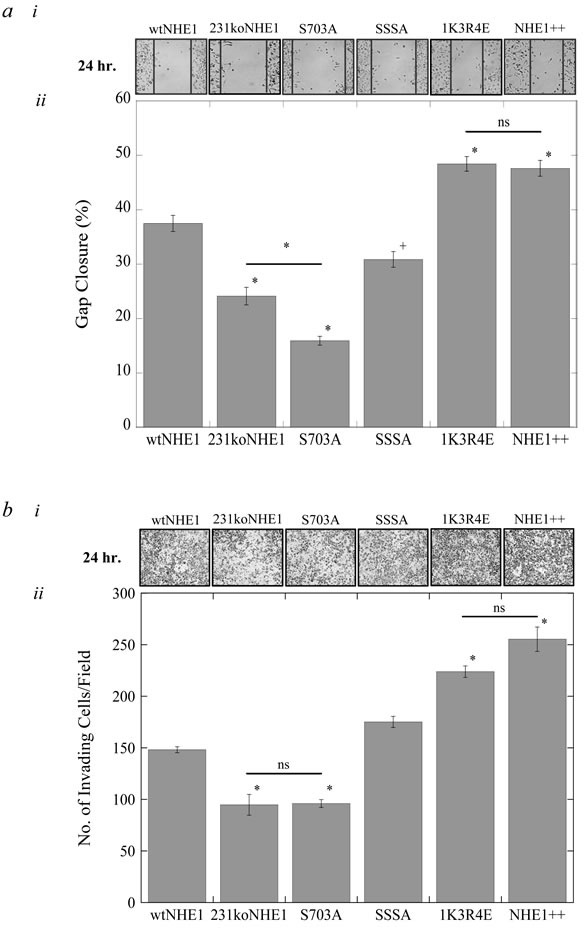
Rates of migration and invasion of mutant NHE1 cells compared to wild-type NHE1-expressing cells and 231koNHE1 cells **a.** Rate of cell migration in mutant NHE1 cells compared to wild-type NHE1 cells and 231koNHE1 cells. Cell migration was assessed using a qualitative wound-healing assay. **i**, Pictorial representation of migration in wtNHE1, 231koNHE1, S703A, SSSA 1K3R4E and NHE1++ cells over time (bright-field microscopy at 10X magnification). **ii**, Rate of closure of the induced gap at 24 hr. in cells cultured in complete growth media [^#^*P* < 0.05, ^+^*P* < 0.01, *P < 0.001; *N* = 4]. **b.** Rate of cell invasion in wtNHE1, 231koNHE1, S703A, SSSA, 1K3R4E and NHE1++ cells over time. Cell invasion was evaluated by quantification of cells traversing through porous Matrigel™-coated transwell inserts over 24 hr. by bright-field microscopy (10X magnification). **i.** Pictorial representation of cell invasion (bright-field microscopy at 10X magnification). **ii.** The number of invading cells per field of mutant NHE1 cells compared to cells expressing wtNHE1 and 231koNHE1 cells [*P < 0.001; *N* = 4].

We examined the effect of mutations to NHE1 regulatory regions on migration and invasion of cells carrying the mutant NHE1 proteins. In S703A-expressing breast cancer cells, there was a marked decrease in the rates of migration and invasion compared to cells expressing wtNHE1 (*P < 0.001, *N* = 4, Figure 4a and 4b). S703A cells were also less migratory than 231koNHE1 cells, and both these cell types had the lowest invasion rates of all the cells studied (^+^*P* < 0.01, *P < 0.001, *N* = 4, Figure 4a and 4b). In cells where the ERK1/2-mediated regulation of NHE1 was disrupted (SSSA), the rate of migration over 24 hr. was also reduced relative to wtNHE1 cells (^+^*P* < 0.01, *N* = 4, Figure 4a), but there was no significant difference in the invasive capacity of these cells (Figure 4b). In contrast, rates of migration and invasion were significantly higher in 1K3R4E cells where NHE1 was rendered constitutively active (*P < 0.001, *N* = 4, Figure 4a and 4b). We hypothesized that 1K3R4E cells might be behaving like cells with a higher NHE1 expression. We therefore examined an MDA-MB-231 cell line containing both endogenous NHE1 protein and exogenous wild-type NHE1 protein (NHE1++). We found that these cells, where wild-type NHE1 was over-expressed, also had increased rates of migration and invasion similar to those observed in cells expressing the 1K3R4E mutant NHE1 protein (Figure 4a and 4b).

### Effect of NHE1 regulatory mutations on colony and spheroid growth

**Figure 5:**
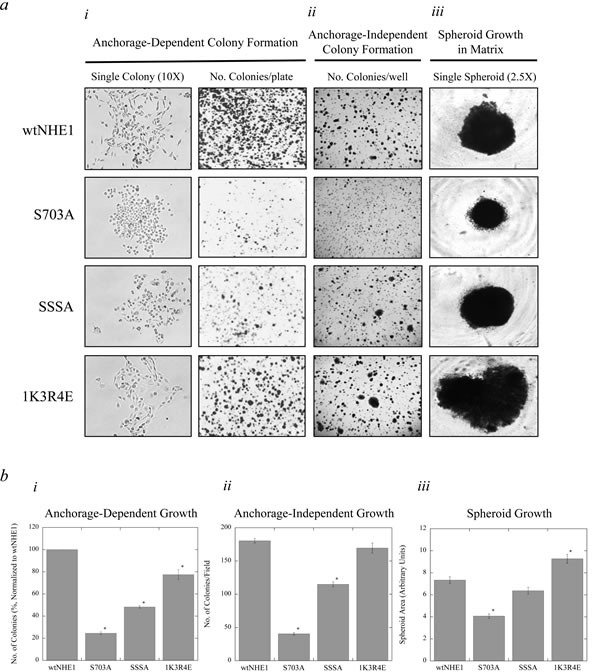
Colony formation and spheroid growth of NHE1 mutant cells compared to wtNHE1 cells Cell colonization was assessed by anchorage-dependent and anchorage-independent (in soft agar) colony forming assays and spheroid growth assays in Matrigel™ matrix (*a* and *b*). **a.i.**, Anchorage-dependent colony formation. Colony growth of wtNHE1, S703A, SSSA, and 1K3R4E cells on cell culture-treated plates was used to evaluate anchorage-dependent growth over 2 weeks, after which cells were fixed, permeabilized and stained with crystal violet. Images shown represent single colony morphology under bright-field microscopy at 10X magnification prior to staining, and the total number of stained colonies per 60 mm plate. **a.ii.** Anchorage-independent colony formation. As described in the Materials and Methods, colony growth of wtNHE1 and mutant NHE1 cells embedded in 0.7% soft-agar in 6-well plates was used to evaluate anchorage-independent cell colonization over 6 weeks. Images shown are a representation of the total number of colonies per well. **a.iii.** Spheroid growth in Matrigel™ matrix. wtNHE1 and mutant NHE1 cells were aggregated, embedded, and grown in a suspension of 5% Matrigel™ in a 96-well plate to determine their ability to form spheroids. After 7 days, cells grow and develop into one single spheroid cell cluster per well. Images shown represent a single spheroid under bright-field microscopy at 2.5X magnification. **b.** Graphical representation of anchorage-dependent and anchorage-independent colony formation and spheroid growth in wtNHE1 and mutant NHE1 cells. Colonies with >50 cells each for anchorage-dependent growth, and colonies with a diameter of >1 mm for anchorage-independent growth, were counted and represented graphically (*b.i* and *b.ii* respectively), as was an arbitrary comparison of the area of spheroid growth of the different cell types (*b.iii*) [*P < 0.001; *N* = 3].

To investigate the ability of mutant NHE1-containing MDA-MB-231 cells to form new metastases *in vitro*, we studied anchorage-dependent colony formation and anchorage-independent embedded colony growth in soft agar and Matrigel™ matrix. Parental MDA-MB-231 cells are aggressively metastatic; likewise, cells expressing wtNHE1 exhibited a high capacity for colony growth both on substrate and in soft agar (Figure 5). In S703A-expressing cells, where stimulation of NHE1 by phosphorylation at Ser^703^ is prevented, colony formation and spheroid growth were markedly reduced (*P < 0.001, *N* = 3, Figure 5a, 5b). S703A cells displayed the least colony-forming growth potential of all mutant NHE1-expressing cell types regardless of anchorage dependency in comparison to wtNHE1. Interestingly, anchorage-dependent colony formation of S703A cells (Figure 5b.i) was even less than that observed in 231koNHE1 cells (Figure 9a.ii), but anchorage-independent colony formation in soft agar of S703A cells (Figure 5b.ii) was comparable to that of 231koNHE1 cells (Figure 9b.ii). SSSA cells also had reduced anchorage-dependent colony formation though the effect was not as pronounced as in S703A cells. Similarly, SSSA-expressing cells also exhibited reduced anchorage-independent colony formation in soft agar, though again the effect was not as obvious as in S703A cells. There was no significant effect of the SSSA mutation on spheroid growth in Matrigel™ matrix. The 1K3R4E-expressing cells had similar anchorage-independent embedded growth in soft agar relative to the wtNHE1 cells but had slightly reduced anchorage-dependent colony formation. In spheroid growth assays, 1K3R4E cells formed significantly larger spheroids (*P < 0.001, *N* = 3, Figure 5a.iii, 5b.iii). Notably, when single colony morphology of anchorage-dependent colony growth was more closely examined, the rounded, epithelial-like phenotype of S703A cells was conspicuous. Whereas wtNHE1, SSSA, and 1K3R4E cells form single colonies with indistinct cell-cell connections, the S703A cells showed much more robust cell-cell contacts (Figure 5a.i).

### Effect of mutations to NHE1 on vimentin and β-catenin expression

**Figure 6:**
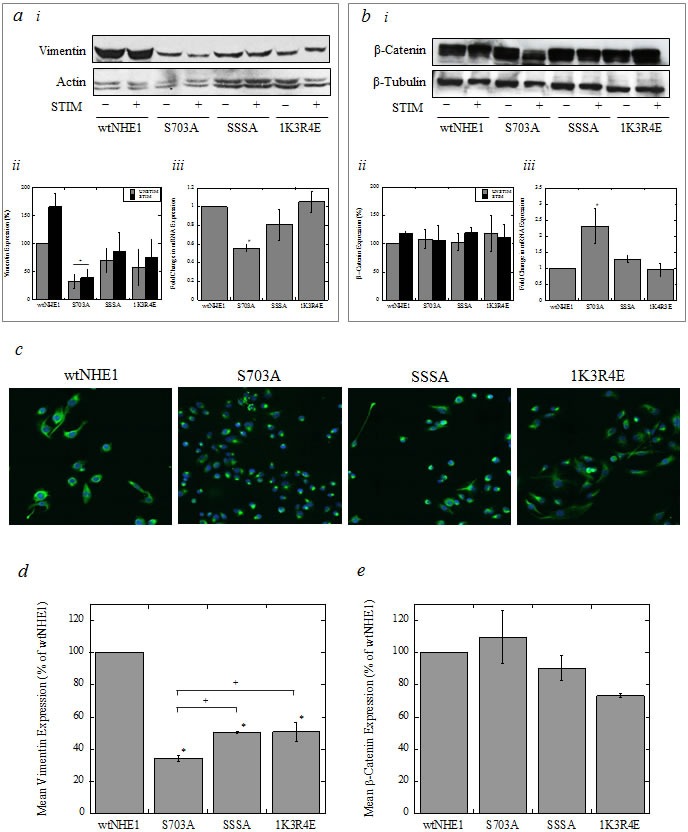
Expression of vimentin and β-catenin in wild-type and mutant NHE1-expressing cells The expression of mesenchymal marker vimentin and epithelial marker β-catenin was assessed by western blot, qRT-PCR, (*a, b*) and laser-scanning cytometry/microscopy (*c-e*). **a.** and **b.** Western blot and qRT-PCR analysis of vimentin and β-catenin in cell lysates of MDA-MB-231 cells expressing wtNHE1, S703A, SSSA, and 1K3R4E. For western blot analysis, cells were cultured in either unstimulated (10% serum in complete media) or stimulated (0.2% serum) conditions for 24 hr. prior to lysis. Actin and tubulin were used as loading controls. a.**i**, Representative western blot showing vimentin expression, and a.**ii**, corresponding quantification of protein expression by densitometry. a.**iii**, mRNA expression of vimentin determined by qRT-PCR. b.**i**, Representative western blot showing β-catenin expression, and b.**ii**, corresponding quantification of protein expression by densitometry. b.**iii**, mRNA expression of β-catenin determined by qRT-PCR. Protein expression of vimentin (*a*) or β-catenin (*b*) expression by western blot was compared with levels in the unstimulated wtNHE1 cell lysates [^+^*P* < 0.01, *N* = 3]. Vimentin and β-catenin expression at the transcriptional level (mRNA) was compared to GAPDH [^#^*P* < 0.05, *N* = 5] in unstimulated cells. **c.** Pictorial representation of vimentin fluorescence (green) and DAPI nuclear staining (blue) in fixed, unstimulated wtNHE1, S703A, SSSA, and 1K3R4E cells by laser-scanning microscopy at 40X magnification. **d.** and **e.** Quantification of vimentin (*d*) and β-catenin (*e*) protein expression in unstimulated conditions as a measure of mean integral fluorescence [^+^*P* < 0.01, *P < 0.001, *N* = 3].

Epithelial-mesenchymal transition (EMT) is considered a key developmental process in tumorigenesis, wherein epithelial cancers like breast cancer become metastatic. We assessed the expression of mesenchymal marker vimentin and epithelial marker β-catenin in cells expressing mutant NHE1 by western blot analysis and laser-scanning cytometry/microscopy. Western blot analysis showed that neither vimentin (Figure 6a) nor β-catenin (Figure 6b) expression in wtNHE1 or mutant NHE1 cells was significantly altered by the presence (UNSTIM) or absence of serum (STIM). However, vimentin protein expression was significantly decreased in S703A cells compared to wtNHE1 cells (^+^*P* < 0.01, *N* = 3, Figure 6a.i and 6a.ii). Interestingly, vimentin and β-catenin expression in MDA-MB-231 cells where NHE1 was knocked out (231koNHE1) was comparable to that of wtNHE1 cells at both protein and mRNA levels (*N* = 3, Supplementary Figure 1c). In SSSA and 1K3R4E cells, assay of vimentin protein expression by western blot analysis revealed a reduction in protein expression that was not statistically significant. The reduction of vimentin expression in S703A cells was also seen at the transcriptional level where mRNA expression was significantly decreased in these cells compared to wtNHE1 cells (^#^*P* < 0.05, *N* = 5, Figure 6a.iii). Expression of vimentin mRNA in SSSA and 1K3R4E cells was comparable to wtNHE1 cells. We further analyzed vimentin expression in mutant NHE1 cells by laser-scanning cytometry/microscopy. Images show vimentin expression (green fluorescence) in S703A, SSSA and 1K3R4E cells compared to wtNHE1 cells (Figure 6c), where mean integral fluorescence is proportional to protein expression. When quantified, vimentin expression was significantly decreased in all NHE1 mutant breast cancer cells (*P < 0.001, *N* = 3, Figure 6d); however, the down-regulation of vimentin observed in S703A cells (^+^*P* < 0.01, *N* = 3, Figure 6d) was greater than that of SSSA and 1K3R4E cells. Additionally, vimentin also appeared to become aggregated in S703A cells, unlike the even cytosolic distribution seen in wtNHE1 cells (Figure 6c). Expression of β-catenin protein, as determined by western blotting (Figure 6b.i and 6b.ii) or laser-scanning cytometry (Figure 6e), was unaffected by mutations to NHE1. However, on the transcriptional level, gene expression of β-catenin was significantly higher in S703A cells (#*P* < 0.05, *N* = 5, Figure 6b.iii). We further examined the mRNA expression of other factors involved in EMT: transcription factors Twist, Snail, and Slug; matrix metalloproteinases MMP2 and MMP9; E-cadherin (epithelial marker) and N-cadherin (mesenchymal marker). MMP2 mRNA levels decreased in S703A-containing cells, while MMP9 expression increased. In SSSA cells, mRNA expression of Twist, Slug, and E- and N-cadherin are increased, while in 231koNHE1 cells, E-cadherin mRNA expression is significantly higher (^#^*P* < 0.05, ^+^*P* < 0.01, *P < 0.001, *N*= 5, Supplementary Figure 2). Western blot analyses to detect the expression of these EMT markers on the protein level were unsuccessful (data not shown); additionally, MDA-MB-231 cells do not express E- and N-cadherin protein. Because of a lack of expression on the protein level, we were unable to draw any conclusions from the changes in mRNA expression of these EMT markers in relation to mutations to NHE1 regulation.

### Effect of BI-D1870 on NHE1 activity and cell proliferation in triple-negative breast cancer cells

**Figure 7:**
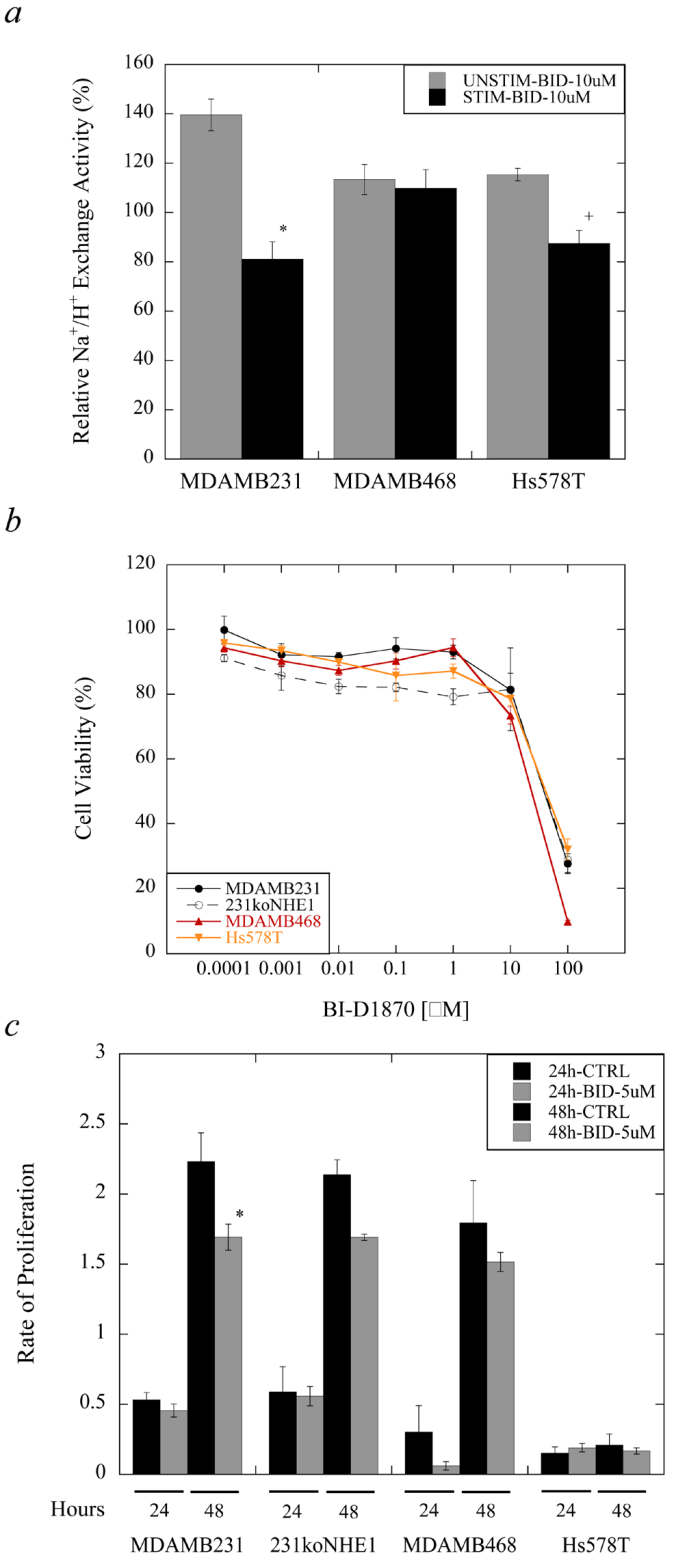
Effect of BI-D1870 on Na^**+**^/H^**+**^ exchange, viability, and proliferation in MDA-MB-231, MDA-MB-468, and Hs578T cells, compared to 231koNHE1 cells BI-D1870, a potent p90^RSK^-specific inhibitor, was tested to evaluate its effects on NHE1 exchanger activity. **a.** Na^+^/H^+^ exchange activity in triple-negative breast cancer cells expressing NHE1 compared to NHE1-knockout cells. Cells were either stimulated by serum deprivation (0.2% serum, STIM) or not (10% serum, UNSTIM) overnight in the presence or absence of 10 μM BI-D1870 prior to measuring NHE1 activity. The rate of Na^+^/H^+^ exchange was calculated from the slope of the first 20 sec. of recovery post-acid load with ammonium chloride and expressed as ΔpH*_i_*/sec. ΔpH*_i_*/sec values measured for cells treated with BI-D1870 were normalized to that of the untreated wtNHE1 control values (not shown) for unstimulated and stimulated cells respectively [*P < 0.0001, *N* = 6]. **b.** Cytotoxicity of BI-D1870 in all cells. Cell viability was determined with MTT assays as described in the Materials and Methods. **c.** Effect of BI-D1870 on cell proliferation. The rate of proliferation of MDA-MB-231, 231koNHE1, MDA-MB-468 and Hs578T cells in response to treatment with 5 μM BI-D1870 was assessed at 24 and 48 hr. Data shown are representative of mean rates of proliferation for each cell type at indicated time points compared to 0 hr. [*P < 0.001; *N* = 3].

The S703A mutation on NHE1 prevents the phosphorylation of Ser^703^ by p90^RSK^, consequently also preventing 14-3-3 binding to the phosphorylated Ser^703^ residue. We therefore wanted to test the potential effect of BI-D1870, a potent and specific inhibitor of p90^RSK^, on Na^+^/H^+^ exchange activity in parental MDA-MB-231 cells. For comparison, we also tested the effect of BI-D1870 on NHE1 activity in two other triple-negative cell lines: the tumorigenic, moderately invasive MDA-MB-468 and the non-tumorigenic, minimally invasive Hs578T cells. In stimulated conditions where NHE1 is activated by serum deprivation (0.2% serum), 10 μM BI-D1870 significantly decreased relative Na^+^/H^+^ exchange in MDA-MB-231 and Hs578T cells, but not in MDA-MB-468 cells. This effect is not observed in unstimulated cells supplemented with 10% serum (^+^*P* < 0.01, *P < 0.001, *N* = 6, Figure 7a). This concentration of BI-D1870 is not cytotoxic (*N* = 3, Figure 7b) so observed effects are not due to cell death. Furthermore, treatment of cells with 5 μM BI-D1870 only over 48 hr. significantly reduced cell proliferation in MDA-MB-231 cells, but had no effect on the proliferation of 231koNHE1, MDA-MB-468, and Hs578T cells (*P < 0.001, *N* = 3, Figure 7c).

### Effect of BI-D1870 on migration and invasion in triple-negative breast cancer cells

**Figure 8:**
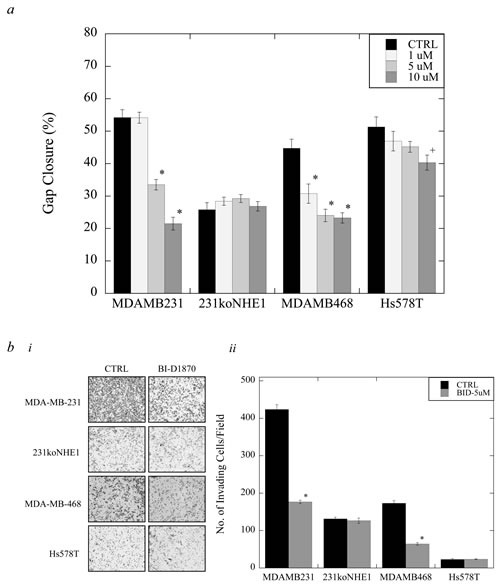
Effect of BI-D1870 on migration and invasion in MDA-MB-231, MDA-MB-468, and Hs578T cells, compared to 231koNHE1 cells Cell migration was assessed by wound-healing assays (*a*), and cell invasion was a measure of the number of cells invading through Matrigel™-coated transwell inserts (*b*). **a.** Effect of BI-D1870 on cell migration. Rate of migration in MDA-MB-231, 231koNHE1, MDA-MB-468, and Hs578T cells treated with 1, 5 or 10 μM BI-D1870 over 24 hr. [^+^*P* < 0.01, *P < 0.001, *N* = 4]. **b.** Effect of BI-D1870 on cell invasion. b.**i**, Pictorial representation of invading cells (bright-field microscopy at 10X magnification). b.**ii**, Rate of invasion of MDA-MB-231, 231koNHE1, MDA-MB-468, and Hs578T cells treated with 5 μM BI-D1870 over 24 hr. [*P < 0.001, *N* = 4].

We examined the effect of BI-D1870 on migration and invasion of MDA-MB-231, 231koNHE1, MDA-MB-468, and Hs578T cells (Figure 8). In MDA-MB-231 cells, a significant reduction in the rate of migration was observed when cells were treated with either 5 or 10 μM BI-D1870 over 24 hr. compared to DMSO-treated controls, whereas in MDA-MB-468 cells, a marked decrease in migration was observed even at 1 μM BI-D1870 (*P < 0.001, *N* = 4, Figure 8a). In Hs578T cells, a significant inhibition of migration was only observed with 10 μM BI-D1870 (^+^*P* < 0.01, *N* = 4, Figure 8a). There was no effect of BI-D1870 on migration in 231koNHE1 cells. Invasion was a measure of cells traversing porous transwell inserts coated with Matrigel™ matrix. When cells were treated with 5 μM BI-D1870 over 24 hr., the number of invading MDA-MB-231 and MDA-MB-468 cells was reduced by more than 50% compared to DMSO-treated control cells (*P < 0.001, *N* = 3, Figure 8b). However, treatment with BI-D1870 did not affect the minimally invasive 231koNHE1 and Hs578T cells.

### Effect of BI-D1870 on colony growth of triple-negative breast cancer cells *in vitro*

**Figure 9:**
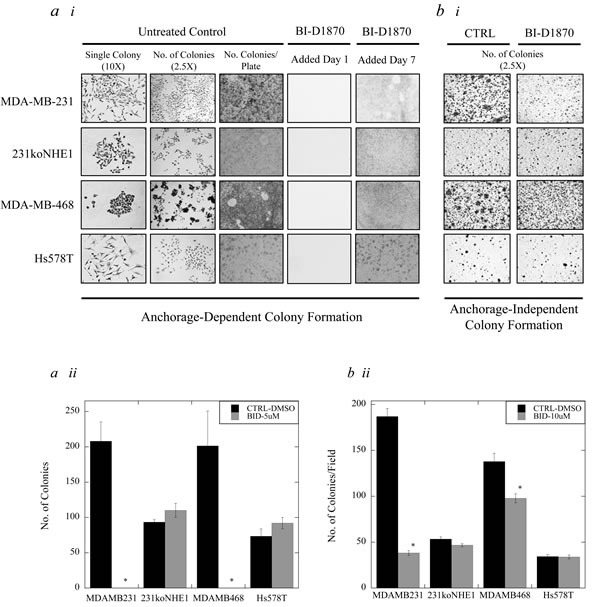
Effect of BI-D1870 on colony growth in MDA-MB-231, MDA-MB-468, and Hs578T cells, compared to 231koNHE1 cells Cell colonization was assessed by anchorage-dependent and anchorage-independent (in soft agar) colony forming assays. **a.** Effect of BI-D1870 on anchorage-dependent colonization. a.**i**, Images shown represent single colony morphology under bright-field microscopy at 10X magnification, multiple colonies at 2.5X magnification, and the total number of colonies per 60 mm plate after staining with crystal violet. 5 μM BI-D1870 was added to cells after seeding either on Day 1 or Day 7. a.**ii**, Graphical representation of anchorage-dependent colony growth of MDA-MB-231, 231koNHE1, MDA-MB-468, and Hs578T cells when 5 μM BI-D1870 was added on Day 7 compared to untreated DMSO controls [*P < 0.001, *N* = 3]. **b.** Effect of BI-D1870 on anchorage-independent colonization. b.**i**, Images show the number of colonies of MDA-MB-231, 231koNHE1, MDA-MB-468, and Hs578T cells formed as a result of anchorage-independent 3D growth in soft agar. Cells in soft agar were treated with 10 μM BI-D1870 in complete media every 5 days and colonies were counted after 6 weeks. b.**ii**, Graphical representation of anchorage-independent soft agar colony growth of cells in the presence of 10 mM BI-D1870 compared to untreated DMSO controls [*P < 0.001, *N* = 5].

Clonogenic assays were used to assess anchorage-dependent colony formation, and 3D colony growth in soft agar was used to evaluate anchorage-independent colony formation, which mimics the formation of new metastases *in vitro*. In clonogenic assays, MDA-MB-231, 231koNHE1, MDA-MB-468 and Hs578T cells were treated with 5 μM BI-D1870 or DMSO in complete growth media over 14 days. When BI-D1870 was added on Day 1 after cells were seeded, no anchorage-dependent colony formation was observed in any of the cell types (Figure 9a.i). However, when BI-D1870 was added on Day 7, after initial cell colonies had established, no further colony growth was observed (of 50 cells or greater per colony) with MDA-MB-231 and MDA-MB-468 cells compared to DMSO controls (*P < 0.001, *N* = 3, Figure 9a.ii). In soft agar assays, 10 μM BI-D1870 was added to complete media to assess anchorage-independent colony growth (Figure 9b). Here, p90^RSK^ inhibition by BI-D1870 decreases anchorage-independent colony growth over 6 weeks in MDA-MB-231 cells and MDA-MB-468 cells (*P < 0.001, *N* = 5, Figure 9b.i and 9b.ii). No effect of BI-D1870 was seen in anchorage-dependent or -independent colony formation of 231koNHE1 and Hs578T cells.

## DISCUSSION

Triple-negative breast cancer remains enormously problematic for treatment, with high recurrence rates that show limited response to chemotherapy [1, 2]. Dysregulated pH homeostasis in these tumor cells plays an important role in facilitating cancer progression and metastasis. It is the perturbed functionality of the Na^+^/H^+^ exchanger NHE1 that is key in the maintenance of the alkaline intracellular pH of tumor cells and the acidic extracellular pH of the tumor microenvironment. Activation of NHE1 occurs through a rightward shift in the pH *vs*. activity curve resulting in higher NHE1 activity at a given intracellular pH [8, 29]. This excessive NHE1 activity promotes extracellular matrix proteolysis and cell invasion [30]. We have previously reported that inhibiting NHE1 activity increases the efficacy of low-dose paclitaxel chemotherapy in TNBC cells, and MDA-MB-231 cells lacking NHE1 expression are unable to establish *in vivo* xenograft tumors in mice [9]. In the present study, we further investigated whether disrupting NHE1 regulation would yield novel insights with which to combat and target metastatic TNBC. We used the MDA-MB-231 TNBC cell model and replaced endogenous NHE1 with wild-type or mutant NHE1 protein expressing three different regulatory mutations. We disrupted NHE1 regulation mediated through Ser^703^ *via* p90^RSK^/14-3-3 (S703A). p90^RSK^, a signal kinase downstream of ERK1/2, activates NHE1 at Ser^703^ in response to serum and growth factors [31, 32]. We also examined the effect of mutation of ERK1/2 phosphorylation sites at Ser^766^, Ser^770^ and Ser^771^ (SSSA). ERK1/2 directly phosphorylates NHE1 at sites including Ser^766^, Ser^770^ and Ser^771^, in response to sustained intracellular acidosis [22, 33]. Finally, we examined the effect of activation of the NHE1 protein through mutation of the high-affinity calmodulin-binding site of NHE1 (1K3R4E). The 1K3R4E mutation also interferes with the auto-inhibitory domain of NHE1, resulting in a constitutively active exchanger [34].

Initially, we characterized the effect of the three mutations on NHE1 expression and activity. We tested the effect of serum deprivation on activity of the protein since, in breast cancer cells, serum deprivation activates the Na^+^/H^+^ exchanger [6, 26]. In MDA-MB-231 cells, preventing the phosphorylation of key residues on NHE1 by ERK1/2 (SSSA) or p90^RSK^ (S703A) prevented NHE1 activation by serum withdrawal (Figure 1b). p90^RSK^-mediated activation of NHE1 by phosphorylation at Ser^703^ is observed in multiple cell types [18, 19]. 14-3-3 binds to the phosphorylated Ser^703^ residue and prevents its dephosphorylation, which is key in the growth factor-mediated activation of NHE1 in response to serum. Mutating Ser^703^ to non-phosphorylatable alanine abolishes this interaction in fibroblasts [20] and lowers basal NHE1 activity in CHO cells, but not in cardiomyocytes [35]. Our results demonstrate that Ser^703^ also plays an important role in NHE1 regulation in TNBC cells though basal exchanger activity was not affected. The SSSA-NHE1 protein was also not activated by serum withdrawal and its basal activity was similarly unaffected. However, this was not reflected in many other phenotypic effector functions in MDA-MB-231 cells expressing this mutation to NHE1. The activity of the 1K3R4E mutant NHE1 protein was also unaltered by serum withdrawal. This may have been due to the constitutive activation of the 1K3R4E-NHE1 protein. We found that the exchanger activity of the 1K3R4E cells was elevated, with it being significantly higher at acidic intracellular pH, akin to exchanger activity in cells over-expressing NHE1 (NHE1++). These results were similar to those reported earlier in which we confirmed that other 1K3R4E-containing cells had elevated NHE1 activity [36].

Analysis of cell proliferation and cell cycle progression indicated that there were significant differences in S703A and 1K3R4E cells, relative to cells expressing wild-type NHE1. S703A cells showed a significant increase in proliferation, which was accompanied by an increase in the proportion of cells in the G1 phase of the cell cycle. The reason for this increase in proliferation is not known at this time but a similar trend was also seen in lung fibroblasts harboring the same S703A mutation to NHE1 [37]. We hypothesize that cell growth and proliferation may be under a different regulatory control than other effector functions, like motility and invasion, which are adversely affected by this mutation to NHE1 as discussed below. The 1K3R4E cells also showed alterations in phases of the cell cycle. We found significantly more 1K3R4E cells in G1, the growth phase associated with increased cell size and biosynthesis activities, and less cells in G2/M, the growth phase leading up to mitotic division. Additionally, the duration of the G2/M phase was shorter and G1 was longer, suggesting that these cells were growing but not dividing (G0 phase). Our results confirm that mutation of these two regulatory regions influence growth of MDA-MB-231 TNBC cells.

The most distinctive change brought about by a mutation to NHE1 was that induced by the replacement of serine 703 with a non-phosphorylatable alanine (S703A). This mutation caused cells to exhibit a more epithelial-like morphology compared to the mesenchymal morphology of cells expressing wild-type NHE1. This finding is significant since cancer-associated epithelial-mesenchymal transition (EMT) is reversible and thought to lend plasticity to tumor cell morphology, allowing for migration and invasion of cancer cells out of the primary tumor and into the bloodstream. The reverse process of mesenchymal-epithelial transition (MET) is considered to be equally important in establishing secondary metastases in organs distant from the tissue of origin [38]. Evidence for the change in phenotype caused by the S703A mutation was gained by examining cell morphology and the cellular expression of mesenchymal marker vimentin. MDA-MB-231 cells normally exhibit a stellate phenotype with an elongated cell body and multiple invasive processes typical of mesenchymal cells [27]. In contrast, S703A-expressing cells were smaller, and more rounded and epithelial-like than wtNHE1 cells. This change in S703A cell morphology correlated with a dramatic down-regulation of vimentin, both at protein and transcriptional levels. Additionally, vimentin distribution became compacted in S703A cells, forming aggregates close to cell nuclei, compared to its uniform cytoplasmic distribution in wtNHE1 cells. While some decrease in vimentin expression was observed in SSSA and 1K3R4E cells, these reductions were less and there was no redistribution of vimentin. Additionally mRNA and total protein levels were unchanged in these cells. Vimentin is a type III cytoskeletal intermediate filament protein and its expression determines cell shape, adhesion, and motility. The changes we found in vimentin were comparable to changes in cell morphology and vimentin expression reported by others in bladder carcinoma cells [39]. This pattern of vimentin expression with associated morphological changes is also seen in another cell type: the non-invasive, hormone receptor-positive MCF-7 breast cancer cells. When MCF-7 cells were microinjected with vimentin or transfected with vimentin cDNA, these rounded, epithelial-like cells adopted elongated shapes similar to those of mesenchymal cells. Conversely, knocking-down vimentin expression in mesenchymal MDA-MB-231 cells resulted in an epithelial-like morphology and disrupted the structural integrity of the remaining vimentin present, causing the formation of small clusters of disorganized filaments close to nuclei [40]. This is strikingly similar to what we observed in S703A cells (Figure 6c). Another more recent study also demonstrated that expressing vimentin in MCF-7 cells increased cell motility and directional migration, while knocking down vimentin in MDA-MB-231 cells decreased rates of migration. More significantly, loss of vimentin expression in these cells also caused cytoskeletal reorganization, and reduced focal adhesions, all of which negatively impacted cell motility [41]. We observed similar decreases in migration, invasion, and colony growth of S703A cells. Overall, the effects of expressing the S703A-NHE1 mutant in MDA-MB-231 cells correlate well with the published effects of loss of vimentin expression on cell phenotype. We also observed reduced vimentin expression in SSSA and 1K3R4E cells when stained with anti-vimentin antibody (Figure 6d), but not to the same extent. Additionally, expression of mRNA and total protein of vimentin in both these NHE1 mutants was not significantly different from wtNHE-expressing cells, whereas S703A cells showed a significant loss of vimentin at both the protein and transcriptional levels. Moreover, SSSA and 1K3R4E cells did not lose the mesenchymal morphology associated with wtNHE1 cells; this was only observed in S703A cells. Serine 703 on NHE1 is the phosphorylation site for p90^RSK^, where 14-3-3 binds to phosphoSer^703^ and prevents its dephosphorylation. Interestingly, 14-3-3 also plays a key role in the dephosphorylation and disaggregation of vimentin by binding to phosphovimentin [42]. We have observed a strong association between 14-3-3 and NHE1 in MDA-MB-231 cells by mass spectrometric (MS) analysis and co-immunoprecipitation (unpublished data). The interaction between NHE1 and vimentin, however, was weaker and fell just under the threshold limits for MS analysis. This raises the intriguing possibility that NHE1 could be tethered to cytoskeletal vimentin intermediate filaments *via* its association with 14-3-3 at Ser^703^. This would then explain why vimentin distribution is so markedly different in S703A cells, where the mutation to NHE1 prevents binding of 14-3-3. It is worthwhile to note that NHE1 is also tethered to the actin cytoskeleton *via* its association with actin-binding proteins ezrin, radixin, and moesin (ERM), which directly bind NHE1 in the region spanning aa 552-560. Mutations to this region of the exchanger resulted in irregular cell shape and disorganization of the cortical cytoskeleton of cells that was independent of ion exchange [43]. Here, we present the first evidence that disruption of NHE1 regulation mediated by p90^RSK^ and, consequently, 14-3-3, affects the expression and cytosolic organization of vimentin intermediate filaments, enough to significantly alter cell morphology and potentially result in mesenchymal-epithelial transition (MET). Surprisingly, the complete knockout of NHE1 did not result in an alteration of cell morphology. The reason for this is unclear at this time; however, it may be that some part of NHE1 activity is necessary for this transition to occur.

To further examine what happens when p90^RSK^-mediated activation of NHE1 in triple-negative breast cancer cells is interrupted, we studied the effect of BI-D1870 on metastatic potential. BI-D1870 is a potent and specific inhibitor of all RSK isoforms. RSK inhibition by BI-D1870 and other small-molecule p90^RSK^ inhibitors has been reported to significantly decrease growth of TNBC cells [44, 45]. Here, we demonstrated that BI-D1870 not only caused a significant decrease in anchorage-dependent and -independent colony growth similar to that seen in the colony growth of S703A cells, but also dramatically decreased the rates of migration and invasion of highly invasive MDA-MB-231, and moderately invasive MDA-MB-468 TNBC cells. BI-D1870 was only minimally effective in the lowly invasive Hs578T TNBC cells. This could be because BI-D1870 also inhibited NHE1 activity in MDA-MB-231 cells and, to a lesser extent, in Hs578T cells. Both are of the claudin-low, basal B subtype of TNBC. However, exchanger activity of MDA-MB-468 cells, of the basal A TNBC subtype, was not affected. Surprisingly, treatment with BI-D1870 appeared to increase the basal NHE1 activity of unstimulated MDA-MB-231 cells; however, the reason for this is not known.

SSSA cells also displayed a lower metastatic capacity, though not to the same extent as S703A cells. Rates of colony formation were significantly reduced in S703A and SSSA cells compared to wtNHE1 and 1K3R4E cells. Anchorage-dependent colony formation is generally indicative of adherent cell growth, while growth in soft agar is a characteristic of anchorage-independent cells that correlates strongly with a more invasive and metastatic cell phenotype [46]. Parental MDA-MB-231 cells are an adherent cell line, so exhibiting anchorage-dependent growth despite having an invasive phenotype is not surprising. All three mutations, however, decreased anchorage-dependent colony formation relative to wtNHE1 cells. A more robust conclusion on anchorage-dependency of mutant NHE1 cells can be drawn from their growth in soft agar. Both wtNHE1 and 1K3R4E cells were capable of establishing colonies in soft agar, larger and more numerous than those seen with either S703A or SSSA cells, suggesting that these cells have a higher metastatic potential. Lower rates of migration were also observed in SSSA cells compared to wtNHE1 cells; however, invasion and spheroid growth were not affected. Invasion and spheroid growth in extracellular matrix are more indicative of *in vivo* conditions, which suggest that the effects we saw on migration and colony formation are less significant in SSSA cells. The SSSA mutation in NHE1 is a change in serine residues 766, 770, and 771 to non-phosphorylatable alanine residues. We have previously shown that one or more of these residues are phosphorylated directly by ERK1/2 and that this affects the response to acute intracellular acid load in various cell types [22, 33, 47]. The ERK1/2 regulatory pathway and amino acid residues 766, 770, and 771, while still having significant effects on the cell's invasive capacity, appears to be of less importance relative to Ser^703^ and the NHE1 auto-inhibitory and calmodulin-binding domain.

1K3R4E-expressing cells exhibited some very significant and interesting changes in behavior, though other traits were not affected by this mutation to NHE1. Cell size, complexity, and the rate of proliferation were not different compared to wtNHE1 cells. Additionally, the amount of anchorage-dependent or -independent colony formation did not increase. However, other parameters that can affect the potential metastatic behavior of cells *in vivo* changed dramatically. For example, the rate of cell migration and invasion through Matrigel™-coated transwell inserts was increased. In order to better evaluate the metastatic potential of these cells, embedded cell growth in Engelbreth-Holm-Swarm mouse sarcoma cell matrix (Matrigel™) was used to simulate the complex, dynamic interplay between cancer cells and the extracellular matrix *in vitro*. This allows for cell growth in three dimensions and the development of spheroid cell masses more similar to the formation of new metastases *in vivo* [27, 48]. The activation of NHE1 in the 1K3R4E cells conferred distinctly larger spheroid growth compared to the wild-type NHE1 protein. Taken together, our data demonstrates, for the first time, that up-regulation of exchanger activity associated with the high affinity calmodulin-binding site is a critical determinant of the metastatic potential of TNBC cells.

We recently reviewed the importance of intracellular ions and NHE1-mediated dysregulation of H^+^ flux in driving cancer progression [49]. In cancer cells, increased intracellular Na^+^ is thought to promote invasiveness [50], while changes in intracellular K^+^ can be correlated with altered rates of proliferation and migration [51]. However, only cytosolic Ca^2+^ signaling has been implicated in inducing epithelial-mesenchymal transition in breast cancer cells [52]. Increased intracellular Na^+^ can occur from elevated cellular alkalinization by NHE1; however, this was not detected in the present study, likely due to the activity of sodium-extruding proteins such as Na^+^/K^+^-ATPase. In an analysis of primary patient tumor proteins, we reported a 1.5 to 2-fold increase in NHE1 mRNA expression in cancers of the breast, particularly of the invasive subtypes, a correlation that was not seen with sodium-bicarbonate transporters or voltage-gated sodium channels [49]. We did not find differences in levels of intracellular Na^+^ or Ca^2+^ between the mutant and wtNHE1 cells; however, S703A cells had significantly elevated intracellular K^+^ levels than wtNHE1 cells (Supplementary Figure 1b). While the reasons for this are not clear, altered K^+^ channel conductance and K^+^ channel expression can affect cell proliferation [51]. Whether altered K^+^ channel activity plays a role in the increased proliferation rates of S703A cells is not known at this time.

In this study, our goal was to investigate the underlying regulatory mechanisms that instigate the dysregulation of NHE1 activity. We examined three regulatory regions of the NHE1 protein: Ser^703^, the group of Ser^766^, Ser^770^ and Ser^771^, and the high-affinity calmodulin-binding site of NHE1 located at amino acids 636-659 [12]. The group of phosphorylatable amino acids on NHE1 at Ser^766^, Ser^770^ and Ser^771^ has a significant but more minor role in affecting MDA-MB-231 cell invasiveness. In contrast, the high-affinity calmodulin-binding site of NHE1 and Ser^703^ have critical roles in regulating metastatic behavior. Ser^703^, in particular, could play a role in maintaining metastatic potential in these cells. The fact that chemical inhibition of p90^RSK^ by BI-D1870 adversely affects the metastatic potential of otherwise invasive TNBC cells lends credence to this hypothesis. We suggest that Ser^703^ on the NHE1 C-terminal may also be an important “phosphorylation switch” regulating EMT in TNBC cells. Phosphorylation of Ser^703^ by p90^RSK^, and maintenance of its phosphorylation state by the binding of 14-3-3 regulatory proteins, may then be involved in maintaining the NHE1 signal scaffold that sustains the invasive mesenchymal morphology of MDA-MB-231 cells *via* its interaction with vimentin intermediate filaments. Dephosphorylation of Ser^703^ (or its mutation to a non-phosphorylatable residue) could thus revert these cells to an epithelial-like phenotype where the metastatic potential inherent to parental cells expressing endogenous wild-type NHE1 is substantially diminished. The Ser^703^ site on NHE1 may thus be a useful and promising new target for treatment of triple-negative breast cancer.

## MATERIALS AND METHODS

### Cell lines and culture conditions

MDA-MB-231 cells, including all mutants made in this parental cell line, and MDA-MB-468 cells, were cultured in high-glucose modified DMEM (HyClone) supplemented with 10% fetal calf serum (HyClone), 10 mM HEPES, and 1000 units/ml penicillin/streptomycin (Gibco) under standard culture conditions (5% CO_2_, 37°C and high humidity). Hs578T cells were grown in similar culture media additionally supplemented with 0.01 mg/ml bovine insulin but without penicillin/streptomycin. Starvation media contained only 0.2% serum but was otherwise identical in composition. NHE1 mutant cell lines were grown in the presence of 400 mg/mL G418 (Geneticin Sulfate, Santa Cruz). All parental cell lines were authenticated by DNA analysis (DDC Medical, Ohio) and showed >95% homology to the ATCC STR profile. BI-D1870 (2-(3,5-Difluoro-4-hydroxyphenylamino)-8-isopentyl-5,7-dimethyl-7,8- dihydropteridin-6(5H)-one; Axon MEDCHEM, Netherlands), a potent and specific inhibitor of p90^RSK^, was prepared in DMSO and used at the indicated concentrations.

### Generation of NHE1-knockout and NHE1-mutant MDA-MB-231 cells

In MDA-MB-231 breast cancer cells, endogenous NHE1 was excised using CompoZr^®^ Zinc Finger Nucleases (Sigma-Aldrich) specifically targeted to knockout the human NHE1 gene *SLC9A1*, according to the manufacturers' protocols. Knockout cells were screened and selected as previously described [9]. NHE1-knockout MDA-MB-231 cells were used to make stable cell lines containing wild-type and mutant NHE1 proteins. The pYN4+ plasmid [53], which contains cDNA for the full-length wild-type human NHE1 protein (wtNHE1), was stably introduced into the NHE1-knockout cells by Amaxa nucleofection (Lonza). The plasmid has a hemagglutinin (HA) tag that does not affect exchanger activity [54]. Site-specific mutagenesis was used on the pYN4+ plasmid to generate NHE1 mutants as described earlier [55]. To determine the effect of various phosphorylation sites on NHE1 activity, one site was individually targeted (Ser^703^), and three other sites were targeted in tandem (Ser^766^, Ser^770^, Ser^771^), where serine residues were altered to alanine, a non-phosphorylatable amino acid residue, to generate the stable S703A and SSSA mutants respectively [22]. Additionally, four positively-charged residues were targeted together (Lys^641^, Arg^643^, Arg^645^, Arg^647^) and replaced with negatively-charged glutamic acid residues to generate the stable 1K3R4E mutant as described earlier. We have previously shown that this precise mutation results in a hyperactive NHE1 protein [13]. For comparative purposes, we also generated an NHE1 over-expression mutant (NHE1++) where the pYN4+ plasmid (wtNHE1) was introduced into the parental MDA-MB-231 cells that also expressed endogenous NHE1 protein.

### Na^+^/H^+^ exchanger activity: Measurement of ion concentration and proton (H^+^) flux

#### Intracellular pH

Intracellular pH (pH*_i_*) was measured *via* fluorescence using the pH indicator dye, BCECF (2′,7′-bis(carboxyethyl)-5(6)-carboxyfluorescerin-acetoxymethyl ester). Briefly, cells were grown to 80% confluence on rectangular glass coverslips in 35 mm dishes in complete media, and either serum-starved or not for 24 hr. post-attachment. BCECF-AM, a cell permeable dye, was added to cells for 20 minutes at 37°C. During this time, cytosolic esterases cleave the AM ester, allowing fluorescent BCECF to become charged, polarized, and cell impermeable; intracellular BCECF fluorescence is thus a measure of pH*_i_*. Cells were then acidified by addition of ammonium chloride (50 mM, 3 min) followed by its rapid withdrawal [54]. For recovery post-acid load, cells were perfused with Na^+^-free and then Na^+^-containing buffers. After measurement of activity, pH*_i_* was calibrated using buffer containing nigericin and high K^+^ at pH 6, 7 and 8. The ratio of BCECF fluorescence with excitation at 440 nm and 502 nm, and emission at 528 nm, was recorded using a PTI Deltascan Illumination System (Photon Technology International, New Jersey, USA). Buffering capacity was calculated and was similar across all cell types (data not shown). NHE1 activity was calculated from the slope of the first 20 sec of recovery of pH*_i_* from acidification (ΔpH*_i_*/sec). Cells were either stimulated (0.2% serum) overnight prior to measuring NHE1 activity, or supplemented with 10% serum (unstimulated). In experiments testing the effect of BI-D1870 on NHE1 activity, cells were additionally treated with 10 μM BI-D1870 overnight. Data were normalized to the wild-type (wtNHE1) control to show relative Na^+^/H^+^ exchange activity between cell types.

#### Proton flux

Proton affinity was a measure of H^+^ flux (*J*_H_^+^). H^+^ flux was calculated as the product of the rate of change of pH*_i_* over time (ΔpH*_i_*/sec) and the buffering capacity of cells (*B* mmol • 1^−1^ • pH unit^−1^) as previously described [56]. Buffering capacity was determined by varying ammonium chloride concentrations (10 mM, 30 mM, or 50 mM) and measuring intracellular pH and calculating buffering as described earlier [56].

#### Ion concentration

Intracellular and extracellular Na^+^, K^+^, Mg^2+^, and Ca^2+^ concentrations of wtNHE1 and NHE1-mutant cells were determined using inductively coupled plasma optical emission spectroscopy (ICP-OES, iCAP 6000, Thermo Scientific, Canada). Cells were seeded in a 12-well plate, grown to confluence, and either stimulated (0.2% serum) or unstimulated (10% serum) overnight. Cells were thoroughly washed in sodium- and potassium-free buffer and then lysed with 0.5 mL of 0.1% Triton X-100 and 0.2% nitric acid overnight at 4°C with agitation, prior to sonication for 1 min as previously described [57]. Samples and buffer blanks were then diluted 100 times in ultrapure deionized water and filtered to remove any particulate matter. For the ICP-OES analysis, the digestion method used was EPA 3051, with nitric acid at a ratio of 5 mL HNO_3_ to 20 mL ultrapure deionized water, using the Xpress Mars Microwave Digestion System (CEM Corp., US).

### Morphology

#### Flow cytometry

Cell size *versus* granularity was evaluated using flow cytometry on live cells. Briefly, adherent cells were allowed to attach to 6-well plates overnight and then trypsinized. Cells were washed with phosphate-buffered saline (PBS, HyClone) supplemented with 1% bovine serum albumin and filtered to reduce cell aggregation prior to analysis by flow cytometry (BD Accuri C6, BD Biosciences, US). Dot plots show side scatter area (SSC-A), a measure of granularity, plotted against forward scatter area (FSC-A), a measure of cell size. Histograms show FSC-A for each mutant cell line (grey) compared to wtNHE1 cells (black). Cells were gated to eliminate dead cells and doublets; all dot plots and histograms were similarly gated. 50,000 events (cells) were recorded for each experiment.

#### Microscopy

Cells were plated on 4-chambered glass slides (Falcon) and allowed to attach overnight in complete growth media before fixing with ice-cold 100% methanol for 15 min at −20°C. Nuclei were stained with Hoechst33342 (NucBlue^®^ Fixed Cell ReadyProbes reagent, Life Technologies) at room temperature for 5 min and cells were visualized with an EVOS FL Cell Imaging System (Life Technologies). Images show cells under bright-field overlaid with Hoechst33342 fluorescence (excitation 360 nm, emission 460 nm) to show nuclear localization using a magnification of 20X. Scale bars represent 50 μm.

### Proliferation and cytotoxicity

Cell proliferation (net growth) and cytotoxicity of BI-D1870 was measured as previously described [9]. Briefly, cells were seeded into 96-well plates at a density of 1 × 10^4^ cells/well for 0 to 48 hr. prior to adding MTT [3-(4,5-dimethylthiazol-2-yl)-2,5-diphenyltetrazolium bromide, Sigma] at a final concentration of 0.5 mg/mL. To assess cytotoxicity over 24 hr., serial dilutions of BI-D1870 were used with concentrations ranging from 0.0001 to 100 μM. To test the effect of BI-D1870 on proliferation, cells were treated with 5 μM of the drug over 48 hr. Colorimetric changes were used to assess the rate of proliferation at 0, 24, and 48 hr., measuring the reduction of yellow tetrazolium MTT to purple formazan by metabolically active cells. Cell proliferation over time is directly proportional to OD values. A BioTek Synergy MX microplate reader (BioTek Instruments Inc.) was used to measure absorbance at 570 nm with background subtracted at a reference wavelength of 630 nm. Data were analyzed with BioTek Gen5 software and are shown as an increase in the rate of cell proliferation over time.

### Cell cycle analysis and expression of epithelial-mesenchymal transition (EMT) markers

Cell cycle changes and expression of vimentin (mesenchymal marker) and β-catenin (epithelial marker) were analyzed using a laser-scanning cytometer (LSC), the iCys Research Imaging Cytometer (CompuCyte/Thorlabs, US). Data and image acquisition and analysis was done using iCys 3.4 Cytometric Analysis Software. For LSC, cells were plated at a density of 8000 cells per well in 100 uL of complete growth media in a black 96-well optical clear-bottom plate (Greiner) overnight at 37°C. Cells were then fixed by adding 8% paraformaldehyde in a 1:1 volume ratio directly to the media in the wells for 15 min. at room temperature. Fixative media was removed and cells were washed with 200 uL of phosphate-buffered saline three times prior to permeabilization with 0.1% Triton X-100 in PBS for 10 mins. Cells were washed three times in PBS before adding blocking buffer (1% bovine serum albumin in PBS-Tween) for 45 min. Cells were incubated with primary antibodies against vimentin (Santa Cruz Biotechnologies) or β-catenin (Cell Signaling Technology) in blocking buffer for 1 hr. at room temperature, and then washed three times with PBS. Alexa488- or Alexa594-conjugated secondary antibodies (Invitrogen) were added to cells for 1 hr. before washing with PBS. To determine progression of cells through the cell cycle, DNA-specific fluorochrome DAPI (4′,6′-diamidino-2-phenylindole; 300 ng/ml in PBS) was added to wells prior to placing plate on computer-controlled stage for analysis by LSC overnight. Laser-scanning microscopy images were obtained at 40X magnification, for a total of 16 fields/well. Field images were automatically generated into a composite image of the entire well. For protein expression, the following parameters were measured for each pre-determined contoured cell event: *Area*, physical area (mm^2^) of the contoured cell; *Integral Fluorescence*, total fluorescence (all pixels) in contoured area; and *Maximum Pixel*, fluorescence of brightest pixel in contoured area, as previously described [58]. Mean integral fluorescence was used to determine vimentin or β-catenin expression in mutant-NHE1 compared to wild-type NHE1-expressing cells. For cell cycle analysis, integral fluorescence is proportional to DAPI-DNA binding and maximum pixel values indicate chromatin concentration [28]; these values were used to determine percentage of cells in each phase of the cell cycle in a univariate analysis. While this single time point measurement does not provide information on cell cycle kinetics, the duration of each phase of the cell cycle was estimated with the following equation: T*_Phase_* = [T_C_ × ln(f*_Phase_*+1)]/ln2, where the duration of the cell cycle or population doubling time (T_C_) for MDA-MB-231 cells is approximately 38 hr. (ATCC), and f*_Phase_* is the fraction of cells in a particular phase of the cell cycle [59]. For expression and cell cycle experiments, statistical tables for each parameter were generated by iCys software and exported for further analysis. Images shown are representative of three independent experiments, done with 3 to 6 replicates each. Cumulative data from all experiments were used to quantify expression and cell cycle analysis.

### Quantitative real time-PCR (qRT-PCR)

RNA was extracted from cells using the RNeasy Minikit (Qiagen) according to the manufacturer's protocols. Total RNA (1 μg) was reversed transcribed to cDNA using iScript RT Supermix (BioRad). Quantitative RT-PCR was performed using the iQ SYBR Green Supermix (BioRad) in a Rotor-Gene RG3000 (Corbett Research) cycler, with 40 cycles per sample. Cycling temperatures were as follows: denaturing, 95°C; annealing, 53°C; and extending, 72°C. The primers used are listed in Supplementary Table 1. Differences in gene expression are presented as fold changes normalized to GAPDH expression. Cumulative data from five independent experiments are shown.

### Western blotting

Confluent whole cells were lysed to generate total protein for separation by 8-10% SDS polyacrylamide gels, transferred onto nitrocellulose membranes, and analyzed with antibodies against: NHE1 (BD Transduction Laboratories), vimentin (Santa Cruz Biotechnologies), or β-catenin (Cell Signaling Technology). β-tubulin (Sigma) or actin antibodies (Santa Cruz Biotechnologies) were used as a loading control. Densitometry using the program Image J (ImageJ 1.48v software, National Institutes of Health, Bethesda, MD) was used to quantify expression of the protein of interest *versus* the loading control.

### Migration and invasion

Rates of cell migration (percent (%) gap closure in wound-healing assays over 24 hr.) and invasion (the number of cells traversing through Matrigel™-coated transwell inserts (8 μm pore size) over 24 hr.) were determined as previously described [9]. To assess the effect of BI-D1870 on migration (at concentrations of 1, 5 and 10 μM) and invasion (5 μM), cells were treated with drug for the duration of the assay (24 hr.).

### Colony and spheroid growth assays

#### Anchorage-dependent colony-forming assays

For anchorage-dependent colony-forming assays we used 60 mm polystyrene dishes that had been coated by the manufacturer to introduce a hydrophilic standard growth surface (Sarstedt, Numbrecht, Germany). To quantify anchorage-dependent colony growth, cells were seeded in the 60 mm dishes at a density of 500 cells per dish in complete growth media under standard culture conditions [60]. To test the effect of p90^RSK^ inhibition, 5 μM BI-D1870 in DMSO was added to cultures dishes for the duration of the assay; an equal volume of DMSO alone was added to otherwise untreated controls. After 14 days, colonies were fixed in ice-cold methanol, stained with crystal violet, washed, and left to air-dry. Colonies (>50 cells/colony) were imaged and counted, and the number of mutant colonies was normalized to, and presented as, a percentage of the number of wtNHE1 colonies formed.

#### Anchorage-independent colony-forming assays

Anchorage-independent growth was assessed using colony-forming assays in DNA-grade soft agar (Thermo Scientific) as described previously [61]. Agar was dissolved in complete growth media and 6-well plates were coated with a 0.5% bottom agar layer. 1 × 10^5^ cells per well were suspended in a 0.7% top agar layer. Complete growth media supplemented with 20% fetal calf serum was added to wells and changed every 3-5 days for 4-6 weeks. In BI-D1870 experiments, growth media contained 5 μM BI-D1870 in DMSO; an equal volume of DMSO alone was added to untreated controls. Colonies >1 mm diameter per well were counted. Data are a representation of three independent experiments done in triplicate.

#### Spheroid growth

A modified version of the protocol described previously [62] was used to study the formation of matrix-embedded single spheroids in 3D culture. Briefly, round-bottom 96-well plates were coated with 1.5% DNA-grade agarose (Thermo Scientific) in serum-free, phenol red-free DMEM (50 mL per well). Agarose was allowed to set at room temperature before wells were seeded with 10,000 cells in phenol red-free complete growth media. To induce cell aggregation, plates were centrifuged at 1000 rpm for 15 min. prior to adding 50 mL of a 10% Engelbreth-Holm-Swarm mouse sarcoma cell matrix solution with an initial protein concentration of 8.1 mg/mL (Matrigel™, BD Biosciences). Spheroids were cultured in the resulting 5% Matrigel™-growth media suspension for 7 days in standard culture conditions. Arbitrary spheroid area measurements were obtained with Image Pro Plus software using a Leica DM IRB microscope (at 2.5X magnification).

### Statistical analysis

All data shown are a representation of 3 or more independent experiments done with 3 to 8 replicates each, and are expressed as means ± SEM. GraphPad Prism 5.0 (GraphPad Software, CA, US) was used for statistical analysis, which was done using the two-way analysis of variance (ANOVA) to compare data between multiple groups, and the one-way ANOVA to compare differences between cell types. Post hoc comparisons were made using Bonferroni's (for two-way ANOVA) and Dunnett's (for one-way ANOVA) multiple-comparison tests. A P value less than 0.05 was considered to be significant. Unless otherwise indicated, data were plotted using KaleidaGraph 4.1 (Synergy Software, PA, US).

## SUPPLEMENTARY MATERIAL FIGURES AND TABLE


