# Supplementary material for: Na+/H+ exchanger NHE1 regulation modulates metastatic potential and epithelial-mesenchymal transition of triple-negative breast cancer cells
Source: Oncotarget. 2016 Mar 31;7(16):21091–113. doi: 10.18632/oncotarget.8520 (PMC5008271; doi:10.18632/oncotarget.8520)
Supplement: Supplementary file 1 [file oncotarget-07-21091-s001.pdf]

Na^+/H^+ exchanger NHE1 regulation modulates metastatic potential and epithelial-mesenchymal transition of triple-negative breast cancer cells

Supplementary Material

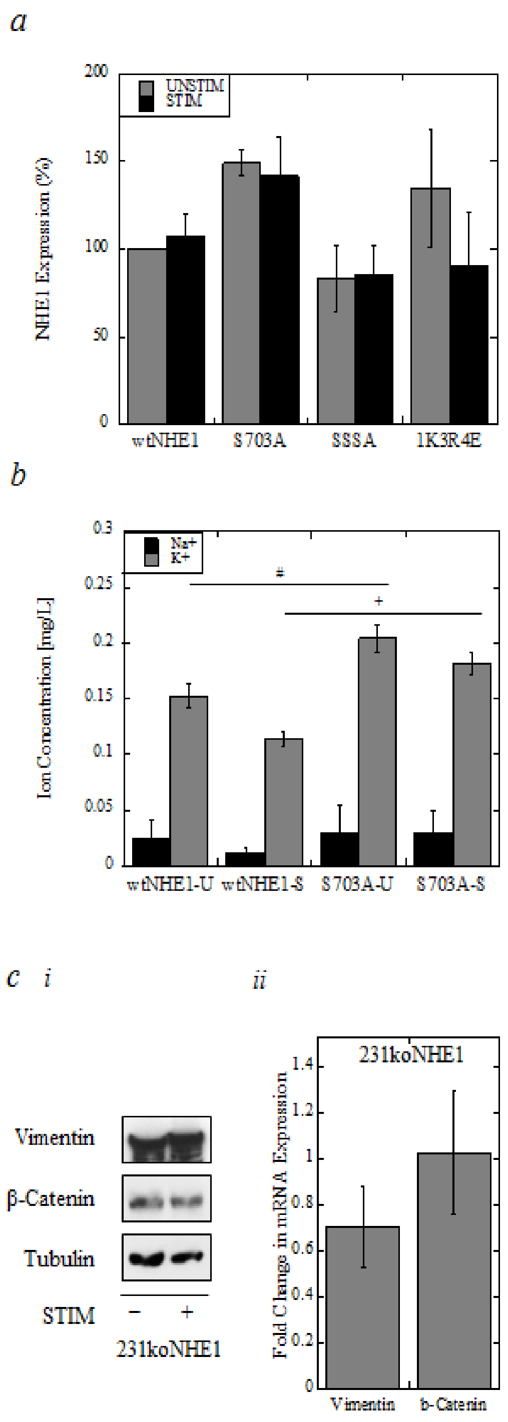

Supplementary Figure 1:

a, Quantification of NHE1 protein expression in wild-type (wtNHE1) and mutant NHE1-expressing cells (S703A, SSSA, 1K3R4E). Cells were either stimulated (0.2% serum, STIM) or not (10% serum, UNSTIM) overnight prior to lysate preparation for western blot analysis. One representative blot of four is shown in Figure 1a (main text). NHE1 protein quantification was estimated by densitometry using Image J software. Data shown are N=4 (with no statistically significant changes in expression).

b, Intracellular Na^+ and K^+ concentration in wtNHE1 and S703A cells. Intracellular cation concentration was assessed in cell lysates prepared as described in the Materials and Methods. Samples were analyzed using inductively coupled plasma optical emission spectroscopy. Data shown indicate changes in intracellular concentrations of K^+, but not Na^+, between wtNHE1 cells and S703A cells in stimulated (0.2% serum, S) or unstimulated (10% serum, U) culture conditions [$^{\#}P<0.05$, $^{+}P<0.01$, N=6].

c, Expression of vimentin and β-catenin in 231koNHE1 cells. ***c.i,*** Representative western blot showing expression of vimentin and β-catenin protein [N=3]. ***c.ii,*** mRNA expression of vimentin and β-catenin as determined by qRT-PCR [N=5].

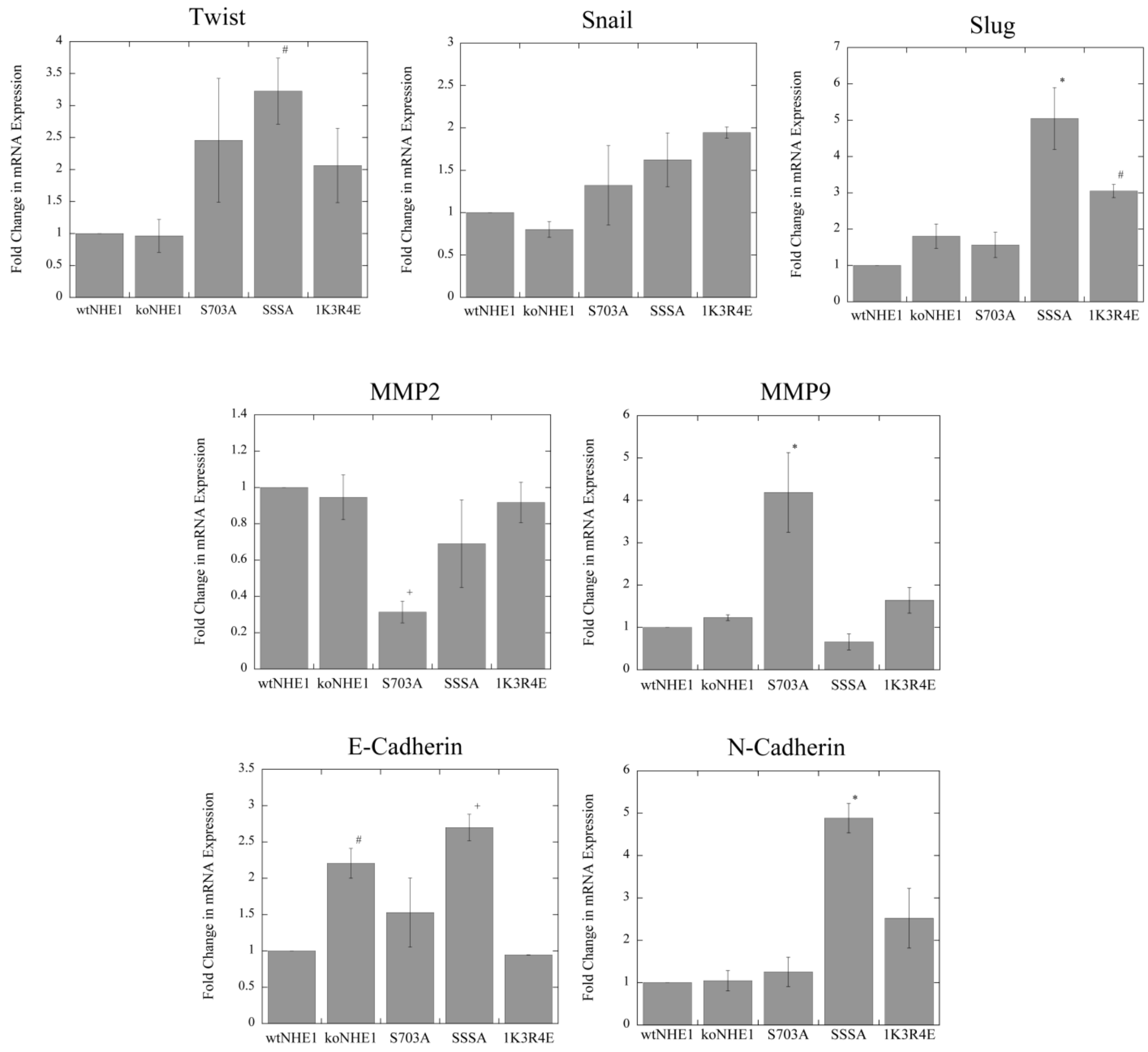

Supplementary Figure 2*:*

Quantification of mRNA expression levels of epithelial-mesenchymal transition (EMT) markers in mutant NHE1 cells compared to wtNHE1 and 231koNHE1 cells. Quantitative RT-PCR was used to determine mRNA expression of transcription factors Twist, Snail, and Slug; matrix metalloproteinases

MMP2 and MMP9; and E-cadherin (epithelial marker) and N-cadherin (mesenchymal marker) [$^{\#}P<0.05$, $^{+}P<0.01$, *P<0.001, N=5].

Supplementary Table 1*:*

List of forward (F) and reverse (R) primer sequences used in qRT-PCR experiments to determine mRNA expression of various markers of epithelial-mesenchymal transition (EMT).

Sequence Name	***Sequence***	***Reference***
GAPDH F<br>GAPDH R	AAG GTG AAG GTC GGA GTC AAC<br>GGG GTC ATT GAT GGC AAC AAT A	Talhouk et al, 2013 [63]
Vimentin F<br>Vimentin R	TCT ACG AGG AGG AGA TGC GG<br>GGT CAA GAC GTG CCA GAG AC	Medici et al, 2008 [64]
b-Catenin R<br>b-Catenin F	GTC CTT CAC TCA AGA ACA AGT AGC<br>GCC AGT AAG CCC TCA CGA TGA TGG	Cheng et al, 2013 [65]
Twist F<br>Twist R	GCA AGC TTA GAG ATG ATG CAG GAC G<br>GAC TCG AGG TGG GAC GCG GAC ATG GA	Wang et al, 2006[66]
Snail F<br>Snail R	ACC ACT ATG CCG CGC TCT T<br>GGT CGT AGG GCT GCT GGA A	Medici et al, 2008 [64]
Slug F<br>Slug R	AGC GAA CTG GAC ACA CAT AC<br>TCT AGA CTG GGC ATC GCA G	Wang et al, 2006 [66]
MMP2 F<br>MMP2 R	ATG ACA GCT GCA CCA CTG AG<br>CTC CTG AAT GCC CTT GAT GT	Sossey-Alaoui et al, 2005 [67]
MMP9 F<br>MMP9 R	AGT TCC CGG AGT GAG TTG AA<br>CTC CAC TCC TCC CTT TCC TC	Sossey-Alaoui et al, 2005 [67]
E-cadherin F<br>E-cadherin R	GAA AGC GGC TGA TAC TGA CC<br>CGT ACA TGT CAG CCG CTT C	Alexander et al, 2006 [68]
N-cadherin F<br>N-cadherin R	TGT TTG ACT ATG AAG GCA GTG G<br>TCA GTC ATC ACC TCC ACC AT	Alexander et al, 2006 [68]
